# COVID-19 Prevention Strategies for Victoria Students within Educational Facilities: An AI-Based Modelling Study

**DOI:** 10.3390/healthcare11060860

**Published:** 2023-03-14

**Authors:** Shiyang Lyu, Oyelola Adegboye, Kiki Adhinugraha, Theophilus I. Emeto, David Taniar

**Affiliations:** 1 Faculty of Information Technology, Monash University, Melbourne, VIC 3800, Australia; 2 Public Health and Tropical Medicine, College of Public Health, Medical and Veterinary Sciences, James Cook University, Townsville, QLD 4811, Australia; 3 Australian Institute of Tropical Health and Medicine, James Cook University, Townsville, QLD 4811, Australia; 4 Department of Computer Science and Information Technology, La Trobe University, Melbourne, VIC 3086, Australia

**Keywords:** COVID-19, epidemiology, infection control, educational facilities, artificial intelligence, deep learning, neural networks

## Abstract

Educational institutions play a significant role in the community spread of SARS-CoV-2 in Victoria. Despite a series of social restrictions and preventive measures in educational institutions implemented by the Victorian Government, confirmed cases among people under 20 years of age accounted for more than a quarter of the total infections in the state. In this study, we investigated the risk factors associated with COVID-19 infection within Victoria educational institutions using an incremental deep learning recurrent neural network-gated recurrent unit (RNN-GRU) model. The RNN-GRU model simulation was built based on three risk dimensions: (1) school-related risk factors, (2) student-related community risk factors, and (3) general population risk factors. Our data analysis showed that COVID-19 infection cases among people aged 10–19 years were higher than those aged 0–9 years in the Victorian region in 2020–2022. Within the three dimensions, a significant association was identified between school-initiated contact tracing (0.6110), vaccination policy for students and teachers (0.6100), testing policy (0.6109), and face covering (0.6071) and prevention of COVID-19 infection in educational settings. Furthermore, the study showed that different risk factors have varying degrees of effectiveness in preventing COVID-19 infection for the 0–9 and 10–19 age groups, such as state travel control (0.2743 vs. 0.3390), international travel control (0.2757 vs. 0.3357) and school closure (0.2738 vs. 0.3323), etc. More preventive support is suggested for the younger generation, especially for the 10–19 age group.

## 1. Introduction

A novel coronavirus, severe acute respiratory syndrome coronavirus 2 (SARS-CoV-2), was identified in December 2019. Due to its high virulence and transmissibility, SARS-CoV-2 spread rapidly around the world over the next two years. By 12 October 2022, 618 million confirmed cases and 6.5 million deaths had been reported worldwide [1]. The first case of COVID-19 in Australia was identified in Victoria on 25 January 2020 and spread significantly in the following years [2]. 

Available reports indicate that children and adolescents appear to be less susceptible to COVID-19 infection [3,4]. Data from the early phase of the COVID-19 pandemic demonstrate that infection among children aged 0–19 years accounted for 2% of the total number of confirmed cases in China, 1.1% in England, and 1% in Italy in 2020. The number of confirmed cases in the United States increased significantly from 2021, accounting for about 13.3% for the age group 0–18 years [3,4,5,6]. To prevent the spread of the virus, it is important to adopt preventive behaviours such as wearing face masks, which have been shown to be effective in suppressing transmission and flattening the pandemic curve [7,8,9,10]. These measures are crucial even after vaccination, as they can increase the suppressive effect [7]. In addition to preventive behaviours, increased testing is essential to prevent the spread of the disease in society. This approach can improve case detection, isolation of infected individuals, and contact tracing [11,12,13]. 

Since 2020, the Victoria government had implemented a series of social restrictions and preventive policies for kindergartens, primary schools, colleges, and universities to prevent COVID-19 infection among students [14]. Schooling in Victoria has changed substantially in the past years, according to research from Monash University [15]. Despite several restrictive measures, the infection rate among children and adolescents in Victoria is still higher than in other areas. The proportion of confirmed cases in people aged under 20 years (25.78%) was more than a quarter of the total number of infections in Victoria from 11 October 2020 to 10 October 2022 [16]. Educational facilities have been reported to contribute significantly to the community transmission of virus, which thrives in populated environments [17]. The reasons for the significantly higher COVID-19 infection rates in Victoria remain unclear. Consequently, it is crucial to identify measures to prevent the spread of the virus in the 0–19 age group, particularly in regions with higher infection rates. A more detailed analysis is therefore needed to understand the local intensity and severity of COVID-19 and its impact on children and adolescents within Victoria. 

With the development of artificial intelligence techniques, deep learning methods are frequently used to simulate epidemiology modelling. The performance of the deep learning approach relies on training data to build the simulation model rather than a pre-defined mathematical model, which avoids unrealistic artificial hypotheses and rules affecting the model’s performance [18]. In this study, we propose an increment learning Recurrent Neural Network-Gated Recurrent Unit (RNN-GRU) model to simulate COVID-19 infection within educational facilities to determine the impact score of risk factors during the infection process. The RNN-GRU is an ideal method to simulate the infection of COVID-19 with a proven continuous learning ability during the training process, considering the different COVID-19 variants. It also presents a reliable performance to avoid the problems of gradient explosion and gradient disappearance in the normal RNN model [19] with a lower computation complexity structure of Recurrent neural networks long short-term memory (RNN-LSTM) [20].

Therefore, this study aims to (1) establish an incremental deep learning RNN-GRU model to simulate COVID-19 infection within educational facilities and (2) examine the impact score of risk factors within educational facilities to provide insight into preventive strategies against COVID-19 infection for the Victoria area.

## 2. Materials and Methods

### 2.1. Study Area and Data Collection

This study was conducted in the greater Melbourne area, Victoria, Australia. Data were assessed from multiple sources. The COVID-19 infection data was extracted from the Victoria government website [16]. This publicly available dataset records all infection case information from 11 March 2020 to 10 October 2022 in Victoria, and includes the date diagnosed, age group, postcode, source of COVID-19 infection, and local government area. The source of COVID-19 infection included four categories: travel overseas, contact with a confirmed case, acquired in Australia, and under investigation. For this study, we analysed all COVID-19 cases in patients aged 0–19 years in Victoria, approximately 240,668 in total, as this age group represents a significant proportion of the student population in educational institutions [21]. The government COVID-19 preventive policy data was sourced from the Coronavirus Victoria website (https://www.coronavirus.vic.gov.au, accessed on 8 December 2022) and the COVID-19 Government response tracker [22,23]. The University of Oxford created the COVID-19 Government response tracker, which has collected COVID-19-related policies since 1st January 2020 and covers more than 180 countries. This study utilised Victoria’s individual COVID-19 preventive policy indicator in the COVID-19 infection simulation model.

### 2.2. Data Pre-Processing

**Figure 1:**
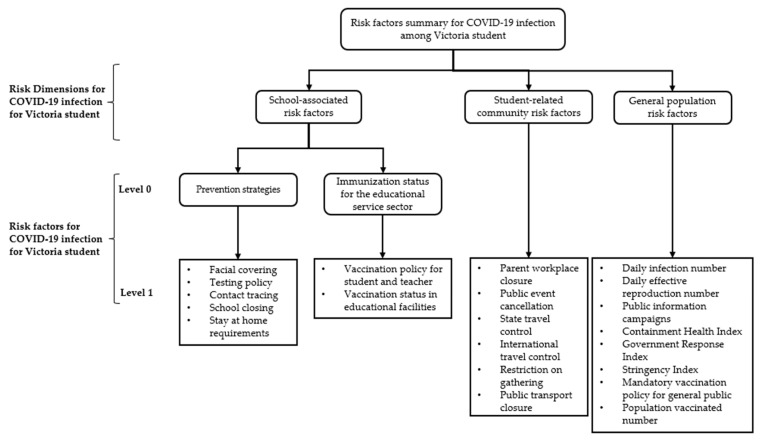
Risk factors classification for preventing COVID-19 infection among Victoria student within education facilities. The school-associated risk factors for COVID-19 infection encompass the various factors and policies in the school environment or initiated by the school that influence the infection among students. The student-related community risk factors refer to the factors and policies that affect the COVID-19 infection among student outside of school time but are still related to students through students’ social gatherings and daily life outside of school time. Lastly, the general population risk factors include total COVID-19 infection case and general government policies for the public within the Victoria area.

To better simulate the COVID-19 infection among Victoria students within educational facilities, the risk that could lead to infection of COVID-19 was categorised into three dimensions: school-associated risk factors, student-related community risk factors, and general population risk factors (Figure 1). It is worth noting that the Victoria Government has implemented a different vaccination policy for the education sector compared to the public [24,25]. All staff in the education sector must be fully vaccinated by 29 November 2022, and a community COVID-19 vaccination programme was launched to improve the vaccination rate among students. Meanwhile, there is no mandatory vaccination policy for the public [24]. Therefore, the vaccination status for the education sector only reflects the vaccination policy and status within the education sector, while the mandatory vaccination policy and the number of vaccinated populations in the general population are risk factors used to measure the progress of vaccination for the public. The detailed risk factor information is presented in Table 1.

**Figure 2:**
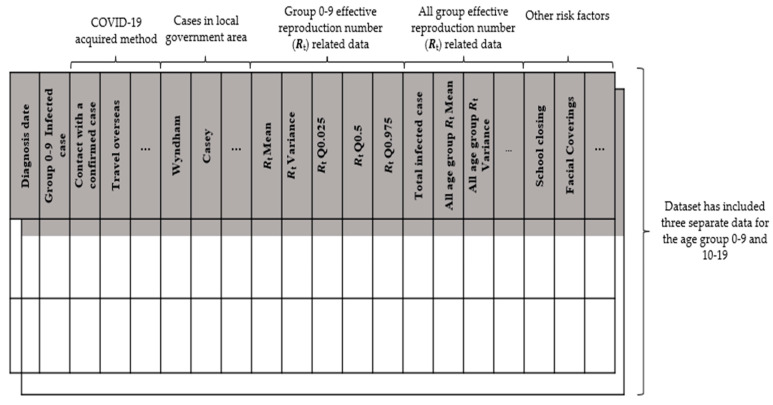
Sample dataset for the COVID-19 infection simulation model. The dataset includes the daily number of infections in that age group, the method of acquisition, the local government area, the effective reproduction number (Rt), and the policy indicator, etc.

For the data pre-processing, the patient data are grouped based on the diagnosis date, age group, and other common attributes. The column-based data indicating the method of COVID-19 acquisition and local government attributes are transformed into the row-based data based on its unique attribute value. The data were further grouped into two separate datasets for age groups 0–9 years and 10–19 years. The effective reproduction number (*R_t_*) analysis was estimated based on the daily infected cases. *R_t_* measures the spreadability of COVID-19 by using the number of human–human transmissions resulting from one infected person [26]. An *R_t_* of 1 means, on average, an infected person will only infect one person. All *R_t_* quantiles (Q0.025, Q0.5 and Q0.975) were also included. Each row in the grouped data represents the COVID-19-infected situation for that day (Figure 2).

### 2.3. Incremental Deep Learning RNN-GRU Model

In this study, we proposed an incremental deep learning RNN-GRU model. The model provides insight into the COVID-19 infection among Victoria’s students within educational facilities and will determine the impact score of risk factors during COVID-19 infection. 

The traditional machine learning training method randomly splits datasets into training and test sets. The training set is used to establish the model and is validated using the testing set. We believe this splitting method is unsuitable for the COVID-19 infection pattern. Firstly, according to the World Health Organization, because of the coronavirus incubation period, symptoms may take up to 14 days to appear [27]. In order words, the number of daily infections of each row in our dataset can impact the infection cases in the following 14 consecutive days. Secondly, there are currently 13 SARS-CoV-2 variants in circulation, including Alpha, Beta, Delta, Gamma, and Omicron. Researchers have proven that the Delta and Omicron have increased infection rates and transmissibility [28,29]. Therefore, the one-off training method is not ideal for creating training and test sets for the COVID-19 infection simulation. 

**Figure 3:**
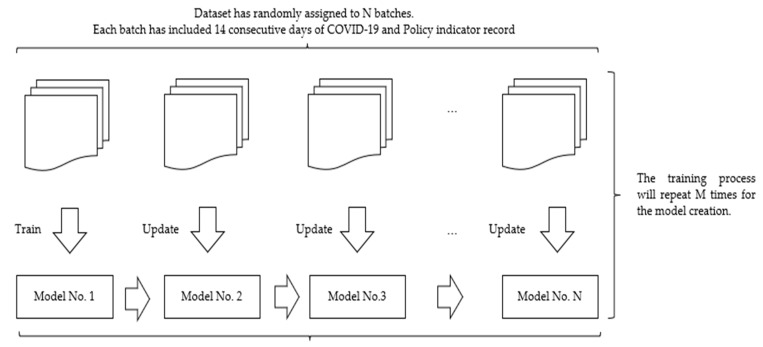
Incremental batch learning method for COVID-19 infection simulation model. Each batch included 14 consecutive days of records. The first batch of data was used to train model 1, while subsequent batches were used to update model parameters.

Therefore, in our proposal model, we have introduced an incremental batch learning method (Figure 3). This method used two separate datasets to represent the age groups 0–9 and 10–19 years. Each dataset was assigned into N small batches randomly. Each batch has included 14 consecutive days of records for model training, which can avoid the impact of random selection in the traditional splitting method. The first batch was used to train the model, and the next N-1 batch was used for the incremental deep learning process to update model parameters. The incremental deep learning process repeated M times to ensure each data point had been involved in the training process. Incremental deep learning continuously updated model parameters to simulate the infection of COVID-19 dynamically. We created 256 small batches for our dataset in the proposed model to keep the CPU at the full workload level.

**Figure 4:**
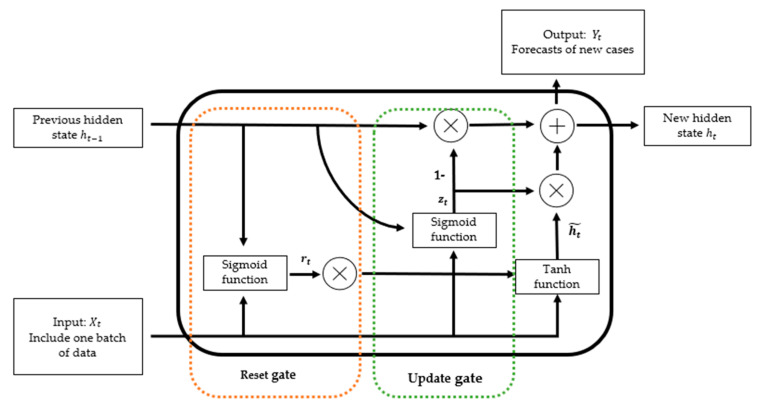
Gate recurrent unit (GRU) structure. The sigmoid function used in the reset gate and update gate of the GRU to decide the amount of information for forgetting and updating. The tanh function is used to calculate the new hidden state *h*_*t*−1_.

Furthermore, the Recurrent Neural Network (RNN) is an ideal method to handle sequential data and is able to memorise previous inputs and involve them in the next model update iteration [30]. As one of the artificial neural networks, the RNN includes three main layers (input layer, hidden layer, and output layer) to connect each other. Each layer includes several neurons to process the data. The neurons are connected by a weighted link, passing processed data from one neuron to another. In traditional deep neural networks, feedback-forward neural networks and convolutional neural networks (CNN) assume that output is independent of the previous input, while the output of RNNs depends on the previous input within the same sequential data. The RNN has taken data from previous input to influence the current input and output during the training process. In this proposal model, we utilised Gated Recurrent Units (GRU) in the RNN to mitigate the impact of short-term memory limits and vanishing gradient problems [19]. The GRU structure contains a reset gate and an update gate. Its output is a combination of the previous hidden state *h_t−1_* and new information from the current input *X_t_*. The reset gate allows the network to retain or forget information from the previous hidden state *h_t−1_* [20] (Figure 4 and Table 2). The value of the reset gate is between 0 and 1, with values close to 0 indicating that the previous hidden state *h_t−1_* should be forgotten, and values close to 1 indicating that the previous hidden state *h_t−1_* should be retained. The update gate determines whether the proportion of new information from the current input *X_t_* should be passed to the hidden state.

### 2.4. RNN-GRU Model Performance Evaluation

To build the RNN-GRU model, the performance of the incremental batch learning process was evaluated by the mean squared error (MSE) to determine the number of repeated times for the incremental learning process.

$$MSE=\frac{{\sum_{i=1}^{n}(Y_{i}-{\hat{Y}}_{i})}^{2}}{n}$$

where $Y_{i}$ represents the observed number of COVID-19 cases in day i, while ${\hat{Y}}_{i}$ is the predicted number of COVID-19 cases in day i, and n is the total number of days.

To measure the simulation performance of the proposed RNN-GRU model, we have compared it with several machine learning algorithms, which were trained by the traditional splitting method. The following performance indices were used to assess the performance of the proposed model; mean absolute error (MAE), root mean squared error (RMSE), and the coefficient of determination (*R*^2^).

$$MAE=\frac{\sum_{i=1}^{n}\left|Y_{i}-{\hat{Y}}_{i}\right|}{n}$$

$$RMSE=\sqrt{\frac{{\sum_{i=1}^{n}(Y_{i}-{\hat{Y}}_{i})}^{2}}{n}}$$

The *R^2^* was used to measure the fitness level of the actual value and predicted value.

$$R^{2}=1-\frac{{\sum_{i=1}^{n}(Y_{i}-{\hat{Y}}_{i})}^{2}}{{\sum_{i=1}^{n}(Y_{i}-\overset{-}{Y})}^{2}}$$

where $Y_{i}$ represents the observed number of COVID-19 cases in day i, $\overset{-}{Y}$ is the mean value of actual COVID-19 cases, while ${\hat{Y}}_{i}$ is the predicted number of COVID-19 cases in day i, and n is the total number of days.

### 2.5. Impact Score Analysis for the Risk Factors 

Despite great simulation ability of RNN-GRU model, there is not a comprehensive understanding method to explain the results of neural networks [31]. To understand the impact of risk factors in preventing the COVID-19 infection among students, we propose a comprehensive impact score examination method in this study, the impact score index.

In the first step, for each risk factor feature $x_{i}$ in the test set $X_{test}$, we perturb that feature by using a random normal distribution (µ = 0 and σ=0.2) to generate a new test set $X_{perturb}$. For the second step, instead of adding noisy data through random normal distribution, each risk factor $x_{i}$ will be randomly shuffled to generate a new test set $X_{shuffle}$. The effect of this perturbation or shuffling is defined by using RMSE. RMSE is a scale-dependent measurement approach but is more sensitive to individual large forecast errors than MAE [12]. In this study, each risk factor impact score is defined as follows:

$${Impact\;Score}_{i}=\sqrt{\frac{{\sum_{i=1}^{n}({{\hat{Y}}_{i_{perturb\;or\;shuffling}}-Y}_{i_{test}})}^{2}}{n}}$$

where $Y_{i_{test}}$ is the predicted value based on the original test set $X_{test}$, and ${\hat{Y}}_{i_{perturb\;or\;shuffling}}$ is the new predicted value based on the new test set $X_{perturb}$ or $X_{shuffle}$. 

To combine the results of the step 1 and 2, a weight attribute was assigned to ${Impact\;Score}_{perturb}$ and ${Impact\;Score}_{shuffle}$ which is defined as follows:

$${Total\;impact\;factor}_{i}=W_{perturb}*{Impact\;factor}_{perturb}+{(1-W}_{perturb})*{Impact\;factor}_{shuffle}$$

## 3. Results

### 3.1. Descriptive Summary

**Figure 5:**
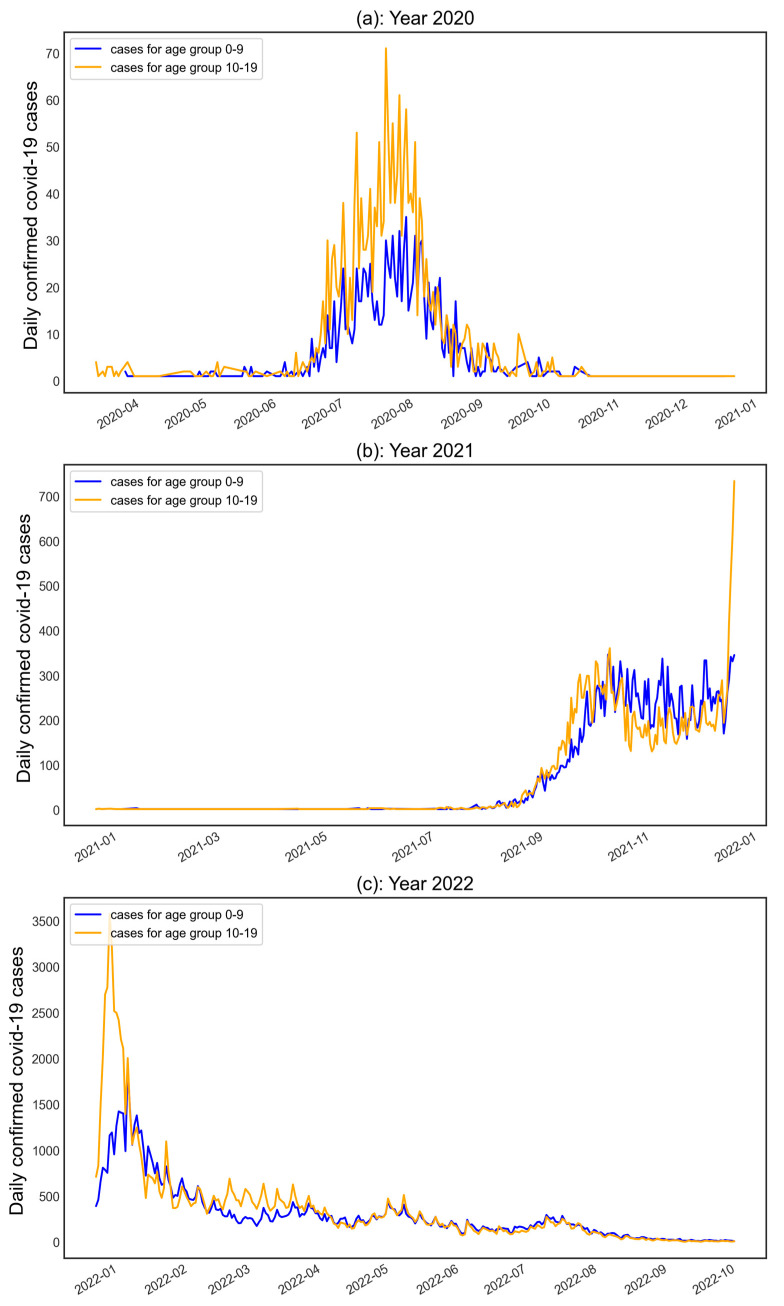
Daily confirmed COVID-19 cases for age groups 0–9 and 10–19 in (**a**) 2020, (**b**) 2021, and (**c**) 2022. The blue line shows the daily cases for the 0–9 age group, while the orange line shows the daily cases for the 10–19 age group.

A total of 943 days’ COVID-19 data for age groups 0–9 and 10–19 in Victoria were used in the study. Figure 5 presents the overall COVID-19 time series data for both age groups in 2020, 2021, and 2022.

In 2020, the daily confirmed COVID-19 cases for the age group 0–9 increased during July and August, reaching a peak at 35 cases on 7 August 2020, while a steeper increase was observed for the age group 10–19 during the same period peaking at 71 cases on 29 July 2020. For 2021, surges in COVID-19 infection mainly happened between September and December, with the highest daily cases of 346 for the age group 0–9 on 20 October 2021 and 734 cases on 31 December 2021 for the age group 10–19. On the other hand, daily confirmed COVID-19 cases presented a significant rise in early 2022, followed by a decreasing trend until October 2022. The number of daily cases reached a record high of 1932 and 3574 cases for age groups 0–9 and 10–19 years in 2022, respectively. Overall, COVID-19 infection among people aged 10–19 years (130,323) was higher than among those aged 0–9 years (110,345).

### 3.2. RNN-GRU Model Performance Evaluation

**Figure 6:**
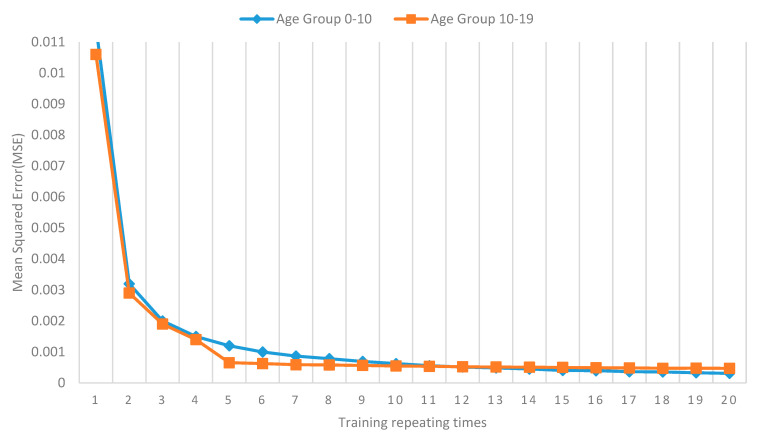
Mean Squared Error (MSE) for incremental batch learning method. The Mean Squared Error (MSE) represents the simulation error of the model for the age groups 0–9 and 10–19, while the training repetitions represent the number of repetitions of the training process used to determine the number of training iterations in the simulation model.

Figure 6 presents the RNN-GRU model training performance during the incremental batch learning process. The infection of COVID-19 among Victoria students for age groups 0–9 and 10–19 has been simulated. With increasing training repeating times, the MSE has decreased gradually for both age groups 0–9 and 10–19 (Figure 4). The MSE remains at the same level after 20 times, which is below 0.001 level for both age groups. Therefore, the proposed RNN-GRU model will establish by repeating incremental training of the COVID-19 dataset 20 times.

To measure the simulation performance of the proposed RNN-GRU model, in this study, we compared RNN-GRU model with several machine learning algorithms, which were trained by the traditional splitting method. Table 3 presents a clear accuracy improvement using the RNN-GRU model for age groups 0–9 and 10–19. Overall, the RNN-GRU model has achieved the best results on all three merits and presents a clear improvement in simulation accuracy.

### 3.3. Impact Score Analysis for Risk Factors of COVID-19 within Educational Facilities

**Figure 7:**
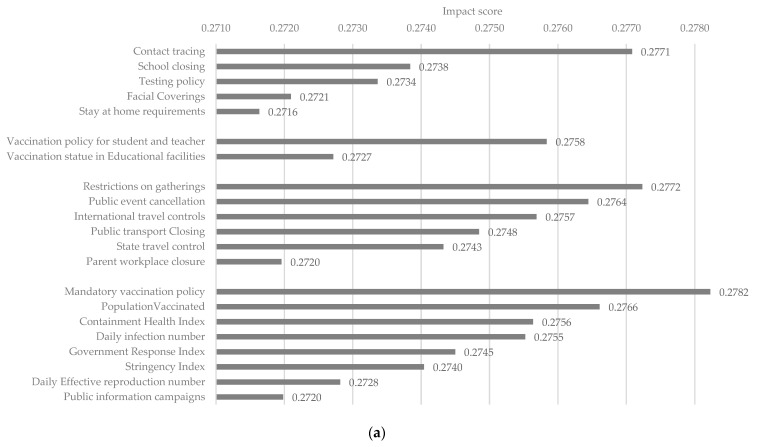

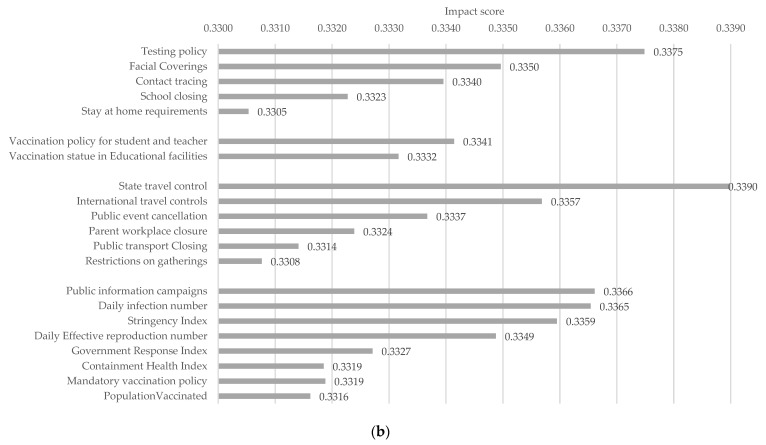
(**a**) Impact score for age group 0–9. (**b**) Impact score for age group 10–19. The impact score is calculated using the proposed comprehensive impact score review method based on the RNN-GRU model to decide the effectiveness of risk factors in preventing COVID-19 infection. The risk factors are listed in descending order within each risk dimension. The school-related risk factors (prevention strategies, immunisation status for the education sector) are listed first, followed by student-related community risk factors, and general population risk factors.

The RNN-GRU model has achieved a great simulation result compared with other machine learning models. In our proposed comprehensive impact score examination method, the $W_{perturb}$ is set as 0.5. Table 4 presents the impact score of dimensions, while Figure 7a,b present the impact score of the risk factor for age groups 0–9 and 10–19, respectively. Of the three dimensions, the general population risk factors have achieved the highest normalised impact score (38.12%) among age groups 0–9 and 10–19, followed by the school-associated risk factors (33.29%). The general population risk factors and school-associated risk factors were significantly higher than the student-related community risk factors (28.59%) (Table 4). For the age groups 0–9 (Figure 7a), the contract tracing (0.2771) that was initiated by the school has contributed the highest normalised impact score in school-associated risk factors, while the testing policy (0.3340) was the most critical factor for age groups 10–19 (Figure 7b). The stay-at-home requirements have shown minor effectiveness among both age groups (0.2727 and 0.332). Within the student-related risk factors in the community, the significance of the risk factors presented between the age groups 0–9 and 10–19 years is different. A different method than the stay-at-home requirement, the restriction on gathering was designed to limit the number of people who could gather in one public place at a given time. This has been found to be the most influential risk factor in the age group 0–9, while it dropped to the least influential factor in the age group 10–19. Meanwhile, the contributions of the state and international travel control increased from the age group 0–9 to 10–19. Parent workplace and public transport closure did not present clear impacts in the analysis. For the general population risk factors, the public information campaigns showed the lowest contribution score in the age group 0–9, while it jumped into the most influential risk factor in the age group 10–19. The opposite change was found in the mandatory vaccination policy, and population vaccinated risk factor. Both risk factors showed a significant contribution in the age group 0–9, while the contribution in the age group 10–19 dropped rapidly. The containment health and government response indexes did not clearly contribute to the impact score analysis.

## 4. Discussion

This study examined the impact score of risk factors of preventing COVID-19 infection within educational facilities in Victoria using an incremental deep learning RNN-GRU model. Compared to other machine learning methods, the model simulation performance was excellent in terms of R^2^, RMSE, and MAE criteria. Employing the incremental batch learning method brings a continuous learning ability to the simulation model and is ideal for handling increasing COVID-19 cases and variants in current society. Three key dimensions were established, namely, school-associated risk factors, student-related community risk factors, and general population risk factors.

The study shows that within the prevention strategies subgroup of the school-associated risk factors, contact tracing initiated by the school and testing policy contributed the most to the total impact score among both age groups. This finding is consistent with previous studies [12,13], which indicated that contact tracing programs and testing policies can be highly effective in swiftly preventing infections on campuses and in the community. As one of the earliest applied preventive strategies, the requirement for facial covering was another contributor to the impact score. Evidence suggests that the use of appropriate facial covering effectively prevent COVID-19 infection by interrupt the transmission of SARS-CoV-2 in both hospital settings and the community [9,10]. One previous study in North-East Nigeria [32] suggests a decreased risk of SARS-CoV-2 transmission among healthcare workers using N95 marks. According to the simulation results presented in Table 4, the impact score associated with school closure appears to be a relatively small contributor to the overall impact of school-related factors in the Victoria region. It has been reported that school closures have limited effectiveness in reducing SARS-CoV-2 transmission and preventing COVID-19 infection compared with other social restrictions [5]. Furthermore, a recent study also found that school closures did not help control the epidemic based on data from the virus outbreak in mainland China, Hong Kong, and Singapore [33]. Specifically, our simulation results revealed that school closure had the third highest impact score within the dimension of preventive strategies for the age group 0–9 years but was not a major contributor for the age group 10–19 years. This finding is consistent with previous research showing that younger children have an increased risk of SARS-CoV-2 transmission than other age groups [34]. Moreover, the simulation results suggest that the vaccination policy for students and teachers has the potential to reduce the incidence of COVID-19 among the student population. Prior clinical evidence demonstrates that COVID-19 vaccination not only prevents severe symptoms of the virus but is also an important tool for reducing the infection rate [35]. Overall, our analysis of impact scores indicates effective contract tracing programs, mandatory face covering requirements, comprehensive testing policies, and vaccination requirements in the education sector have a significant association with the prevention of COVID-19 infection in educational settings. Additionally, school closure presents a different effectiveness level in preventing SARS-CoV-2 transmission between students in age groups 0–9 and 10–19.

For the general population risk factors, public information campaigns are an unignored risk factor. The public information campaign ensured that students were aware of the pandemic virus and accepted COVID-19 vaccines [36]. Its contribution increased significantly in the age group 10–19. A study in Saudi Arabia found a noticeable difference regarding knowledge of COVID-19 prevention between different ages of students through secondary school and university participants [37]. The government stringency index is the measurement indicator for government COVID-19 social restrictions. Social restriction effectively slowed the spread of the virus, as evidenced by the increase in infection doubling time [38]. The daily infection number and mandatory vaccination policy significantly contributed to the total impact score. 

Lastly, the student-related community risk factors focus on the factors and policies that affect the COVID-19 infection rate among students outside of school time but are still related to students through students’ social gatherings and daily life. The state and international travel control contributed the highest impact score. The previous travel control in Bhutan, South Asia [39], indicated that stringent border control and in-country travel had prevented direct entry and widespread transmission of SARS-CoV-2 in Bhutan. However, the contribution of state and international travel control in the age group 0–9 was much lower than in the age group 10–19. Furthermore, common social restriction policies, including restrictions on gatherings and public events were reported to effectively reduce public mobility and infection rate by slowing the spread of SARS-CoV-2 [40]. Based on the analysis of the general population risk factors, and student-related community risk factors, our study suggests that appropriate COVID-19 public information campaigns, travel control, and social restrictions have a significant association with reducing COVID-19 infection rates in educational settings by slowing the spread of SARS-CoV-2. However, social restriction could lead to social and emotional loneliness and mental health issues among students, which needs to be managed [41].

There are a few limitations to this study. Firstly, although the COVID-19 confirmed patient data collected was from the Victoria government website, considering the numerous unreported and asymptomatic cases, these data may not be representative of the actual COVID-19 confirmed cases in Victoria. Secondly, the results of our study were based only on data collected in Victoria, which may limit their generalisability to other contexts. We did not consider risk factors and cases of infection outside the Victoria region, which may affect the external validity of our findings. Therefore, the results may not be applicable elsewhere.

## 5. Conclusions

In summary, a COVID-19 infection simulation analysis using the incremental deep learning RNN-GRU model, based on the dataset from 11 March 2020 to 10 October 2022, was conducted to evaluate the impact score of risk factors for COVID-19 infection within educational facilities in Victoria. The incremental batch learning method has proven a continuous learning ability during the training process, which will be an ideal training method to simulate COVID-19 infection considering the different infection rates of SARS-CoV-2 variants. An RNN-GRU model has provided a reliable simulation performance in the sequential data compared with other machine learning models in the performance analysis. 

The simulation results indicate that the general population and school-associated risk factors have contributed significantly to COVID-19 infection simulation process within educational facilities for age groups 0–9 and 10–19. For the school-associated risk factors, the simulation results showed a significant association between school-initiated contact tracing, face covering requirements, testing policies, and vaccination requirements with COVID-19 infection and the slowing of SARS-CoV-2 transmission within educational facilities. Moreover, within the student-related community risk factors, international and state travel policies and mandatory vaccination policies could effectively prevent direct entry and widespread transmission of SARS-CoV-2 for both age groups 0–9 and 10–19.

Based on the simulation results, it is recommended to establish an effective and swift contact tracing and test system within educational facilities to prevent further transmission of SARS-CoV-2. Wearing facial coverings within educational facilities is highly recommended. It is one of the cost-effective methods to reduce the infection rate by slowing the spread of SARS-CoV-2. Finally, our data analysis highlights the need for increased prevention support for the younger generation in Victoria, particularly the 10–19 age group, as a higher incidence of COVID-19 infections was identified in the period 2020–2022.

## Figures and Tables

**Table 1:** Data dictionary for risk factors of COVID-19 transmission within educational facilities.

Dimensions	Risk Factors	Description
School-associated risk factors (Prevention strategies)	Facial covering	Facial covering requirement
	Testing policy	Testing policy of a symptomatic person
	Contact tracing	School-initiated contact tracing following a positive diagnosis
	School closing	School operation status: open or closed
	Stay at home requirements	The policy that instructs people to remain at home and not leave for non-essential purposes
School-associated risk factors (Immunisation status for the educational service sector)	Vaccination policy for student and teacher	Policies for vaccine delivery of the educators in primary and secondary schools, tertiary education students, and staff
	Vaccination status in educational facilities	Vaccination status for the educators in primary and secondary school, tertiary education students and staff
Student-related community risk factors	Parent workplace closure	Parent or relative’s workplace operation status: open or closed
	Public event cancellation	Public events cancelling status. This factor could lead to a higher secondary infection within the educational facilities
	State travel control	Restrictions on internal movement within the state
	International travel control	Restrictions on international travel
	Restrictions on gatherings	Social gathering restriction level: limit the number of people who can gather in one public place at a given time
	Public transport closing	Operation status: open or closed. Student commute to school by public transport, which may infect them with COVID-19 and lead a secondary transmission within the educational facilities
General population risk factors	Daily infection number	Daily infection number within the Victoria area
	Daily effective reproduction number	Spreadability of COVID-19
	Public information campaigns	COVID-19 public information campaign
	Containment Health Index	Government containment health policy index
	Government Response Index	Government COVID-19 Response Index
	Stringency Index	Social restriction level
	Mandatory vaccination policy	Mandatory vaccination policy for the general public
	Population vaccinated number	The number of vaccinated people in Victoria

**Table 2:** Gate recurrent unit (GRU) formula.

GRU Component	Formula	Function
Reset gate	$r_{t}=\sigma (W_{r}*[h_{t-1},X_{t}])$	Sigmoid function will transfer value into range of 0 to 1 to decide proportion of data for forget purpose
Update gate	$Z_{t}=\sigma (W_{z}*[h_{t-1},X_{t}])$	Sigmoid function will transfer value into range of 0 to 1 to decide proportion of data for update purpose
Output	$\tilde{h_{t}}=tanh\left(W*\left[r_{t}*h_{t-1},X_{t}\right]\right)$ $h_{t}=\left(1-Z_{t}\right)*h_{t-1}+Z_{t}*\tilde{h_{t}}$ $Y_{t}=\sigma (W_{o}*h_{t})$	Update output-based results from reset and update gate

**Table 3:** Results of model evaluation for groups 0–9 and 10–19.

Model	0–9 Years			10–19 Years		
	R^2^	RMSE	MAE	R^2^	RMSE	MAE
RNN-GRU	0.9796	0.2777	0.0601	0.9796	0.2809	0.0634
Kernel Ridge	0.8649	0.3795	0.0965	0.9461	0.3256	0.0853
Ridge regression	0.7879	0.4249	0.1446	0.9244	0.3543	0.1130
Bayesian Ridge	0.7818	0.4279	0.1380	0.9223	0.3568	0.1202
Gradient Boosting Regressor	0.7538	0.4410	0.1622	0.9161	0.3637	0.1181
Lasso regression	0.7478	0.4437	0.1508	0.9156	0.3643	0.1241
ElasticNet	0.7415	0.4464	0.1524	0.8781	0.3992	0.1498
XG Boost Regressor	0.7409	0.4467	0.1512	0.8735	0.4029	0.1221
Random forest	0.3751	0.5567	0.2781	0.7233	0.4901	0.1963
Support vector regression	0.2311	0.5863	0.2956	0.7089	0.4963	0.2047

**Table 4:** Impact score of dimensions for age groups 0–9 and 10–19.

Dimensions	Risk Factors	Age Group		Total	
		Group 0–9	Group 10–19	Sum of Risk Factors	Sum of Dimensions
School-associated risk factors (Prevention strategies)	Contact tracing	0.2771	0.3340	0.6110 ^1^	3.0372 (23.77%) ^2^
	Testing policy	0.2734	0.3375	0.6109	
	Facial Coverings	0.2721	0.3350	0.6071	
	School closing	0.2738	0.3323	0.6061	
	Stay at home requirements	0.2716	0.3305	0.6022	
School-associated risk factors (Immunisation status for the educational service sector)	Vaccination policy for student and teacher	0.2758	0.3341	0.6100	1.2159 (9.52%) ^2^
	Vaccination statue in educational facilities	0.2727	0.3332	0.6059	
Student-related community risk factors	State travel control	0.2743	0.3390	0.6133	3.6534 (28.59%)
	International travel controls	0.2757	0.3357	0.6114	
	Public event cancellation	0.2764	0.3337	0.6101	
	Restrictions on gatherings	0.2772	0.3308	0.6080	
	Public transport Closing	0.2748	0.3314	0.6063	
	Parent workplace closure	0.2720	0.3324	0.6043	
General population risk factors	Daily infection number	0.2755	0.3365	0.6121	4.8714 (38.12%)
	Mandatory vaccination policy	0.2782	0.3319	0.6101	
	Stringency Index	0.2740	0.3359	0.6100	
	Public information campaigns	0.2720	0.3366	0.6086	
	Population Vaccinated	0.2766	0.3316	0.6082	
	Total Effective reproduction number	0.2728	0.3349	0.6077	
	Containment Health Index	0.2756	0.3319	0.6075	
	Government Response Index	0.2745	0.3327	0.6072	

^1^ The risk factors within the dimension are in descending order based on total impact score; ^2^ Normalised total impact score of dimensions. Note: Total normalised risk factor of School-associated risk factors is 33.29%.

## Data Availability

The data for this study are available from the Victoria government website (https://www.coronavirus.vic.gov.au/victorian-coronavirus-covid-19-data, accessed on 8 December 2022).
